# Long-Term Outcomes in Adult Patients with Tick-Borne Encephalitis in Latvia

**DOI:** 10.3390/pathogens15070672

**Published:** 2026-06-25

**Authors:** D. P. Grosa, D. Zavadska, Z. Freimane, L. Karele, G. Karelis

**Affiliations:** 1 Neurology and Neurosurgery Department, Rīga Stradiņš University, LV-1007 Riga, Latvia; guntis.karelis@aslimnica.lv; 2 Department of Neurology and Neurosurgery, Riga East University Hospital, LV-1038 Riga, Latvia; 3 Department of Pediatrics, Rīga Stradiņš University, LV-1007 Riga, Latvia; dace.zavadska@rsu.lv (D.Z.); zane.freimane@rsu.lv (Z.F.); 4 Children’s Clinical University Hospital, LV-1004 Riga, Latvia; 5 Faculty of Medicine, Rīga Stradiņš University, LV-1007 Riga, Latvia; 058407@rsu.edu.lv; 6 Research Group of the Tenured Professor in Neuroimmunology at the Department of Biology and Microbiology, Faculty of Medicine, Rīga Stradiņš University, LV-1007 Riga, Latvia

**Keywords:** tick-borne encephalitis, post-encephalitic syndrome, sequelae, neurocognitive impairment, meningoencephalitis, prognosis, cohort study, arboviral infections

## Abstract

Background: Tick-borne encephalitis (TBE) is an endemic neuroinfectious disease prevalent in parts of Europe and is often associated with persistent neurological and cognitive sequelae. The aim of this study was to evaluate the long-term outcomes and predictors of post-encephalitic sequelae in adult patients with TBE in Latvia. Methods: A retrospective cohort with prospective follow-up was used that included 105 adult patients hospitalized with laboratory-confirmed TBE between 2018 and 2024. The patients’ clinical and demographic data were extracted from medical records, and reassessments were performed ≥6 months after discharge using structured clinical and neurological evaluations for neurocognitive, subjective, and neurological sequelae. Disease severity was classified using the *Mickienė* and *Bogovič* criteria, and sequelae severity was defined according to the Bohr criteria for post-encephalitic syndrome (PES). Results: Sequelae were observed in 52/105 (49.5%) patients and were more frequent in meningoencephalitis than in meningitis cases (18/25 [72.0%] vs. 33/77 [42.9%]). The most common persistent symptoms were impaired concentration (33/52 [63.5%]), fatigue (29/52 [55.8%]), and sleep disturbances (21/52 [40.4%]). Neurological sequelae included tremor (23/52 [44.2%]), vertigo (11/52 [21.2%]), and hearing impairment (5/52 [9.8%]). According to the Bohr criteria, most of the patients had mild sequelae (42/52 [80.8%]), while 10/52 [19.2%] had moderate sequelae; no severe cases were observed. In the multivariable analysis, increasing age was independently associated with greater sequelae severity (OR = 1.045 per year; 95% CI, 1.015–1.073; *p* = 0.003). Sex, comorbidities, biphasic disease, and length of hospital stay were not significant predictors. Acute neurological manifestations, particularly paresis (*p* = 0.002) and tremor (*p* = 0.019), were associated with worse outcomes. Although the disease severity scores correlated with sequelae in unadjusted analyses, neither the *Mickienė* nor the *Bogovič* classification independently predicted outcomes after adjustment. Conclusions: Nearly half of the hospitalized patients with TBE included in this study developed long-term sequelae, which were predominantly neurocognitive and mild in severity. Age was the primary independent predictor of worse outcomes, while acute neurological deficits such as paresis and tremor also indicated increased risk. These findings highlight the substantial burden of post-encephalitic syndrome and the need for structured long-term follow-up in TBE survivors.

## 1. Introduction

Tick-borne encephalitis is a viral infection of the central nervous system caused by the tick-borne encephalitis virus, a member of the *Flaviviridae* family, which is most commonly transmitted by infected ticks but is occasionally acquired through alimentary exposure, such as the consumption of unpasteurized dairy products [1]. This condition represents a significant public health concern in many parts of Europe, particularly in endemic regions, where incidence has increased in recent decades. In Latvia, the highest average 10-year incidence was observed from 1990 to 1999 (27.9 cases per 100,000 population; range: 4.6–53.0), and the average 10-year incidence of TBE from 2007 to 2016, using the TBE case definition, was 9.6 cases per 100,000 population (range: 5.8–14.6). The officially reported incidence of TBE decreased by about 40% after implementation of the E-CDC case definition (2011: 19.4 cases per 100,000; 2012: 11.5 cases per 100,000). The highest yearly incidence after introduction was observed in 2010 (14.6 cases per 100,000), followed by significant decreases in 2014 (7.0 cases per 100,000; *p* < 0.001) and 2015 (6.7 cases per 100,000). While the annual incidence generally decreased, it increased in 2016 (10.9 cases per 100,000) compared with the prior year [2]. Infection can result in a wide spectrum of clinical manifestations, ranging from mild febrile illness to severe meningoencephalitis with potentially life-altering neurological complications [3]. Importantly, a substantial proportion of adult patients experience long-term sequelae, contributing to the overall burden of the disease, with studies indicating that approximately 20–40% (depending on the cohort and follow-up duration) report persistent neurological or cognitive symptoms following TBE [4,5,6,7,8].

The clinical course of TBE comprises asymptomatic infection, symptomatic TBE, and confirmed neurological TBE. The biphasic pattern occurs only in a proportion of symptomatic patients, with an initial nonspecific phase followed by neurological involvement in a subset of patients [8,9]. Although the acute phase has been well described, increasing attention has been directed toward post-encephalitic syndrome, which encompasses persistent neurocognitive, psychiatric, and neurological symptoms that can significantly affect quality of life [4,5,6,8]. These sequelae can occur even in patients with relatively mild disease, raising concerns about under-recognition and insufficient long-term management [4,5,6,7].

Recent research has focused on identifying predictors of disease severity and long-term outcomes; however, the findings remain inconsistent. Older age has frequently been reported to be a risk factor for a worse prognosis, while the roles of sex, clinical form (meningitis vs. meningoencephalitis), and laboratory parameters such as cerebrospinal fluid (CSF) pleocytosis remain controversial. Some studies suggest that more severe acute neurological involvement is associated with higher rates of sequelae, while others have not confirmed this association after adjustment for confounding factors [4,5,6,7]. Furthermore, the discrepancies between objective functional outcomes and patient-reported symptoms highlight the complexity of assessing recovery after TBE [4,5,6].

A better understanding of the burden and predictors of sequelae is essential for improving patient management, guiding follow-up strategies, and informing public health interventions, including vaccination policies.

The aim of this study was to evaluate the clinical characteristics, long-term outcomes, and predictors of sequelae in patients with TBE. Our findings demonstrated that nearly half of the patients examined developed persistent symptoms, predominantly affecting cognitive function, and identified increasing age as the main independent predictor of greater sequelae severity. These results underscore the substantial impact of post-encephalitic syndrome and highlight the need for enhanced long-term care and follow-up of affected individuals.

## 2. Materials and Methods

### 2.1. Study Design and Population

This retrospective cohort study with prospective follow-up included 105 adult patients diagnosed with TBE who were admitted to university and regional hospitals in Latvia between 2018 and 2024. TBE diagnosis was established according to the European Center for Disease Prevention and Control (ECDC) case definition and was confirmed by detection of TBE-specific antibodies using an enzyme-linked immunosorbent assay (ELISA, Germany).

Inclusion criteria were adults (≥18 years) with laboratory-confirmed TBE, hospitalized between 2018 and 2024, with available contact information for follow-up assessment scheduled at ≥6 months after hospital discharge. Exclusion criteria were age <18 years and cases without laboratory confirmation of TBE.

**Figure 1:**
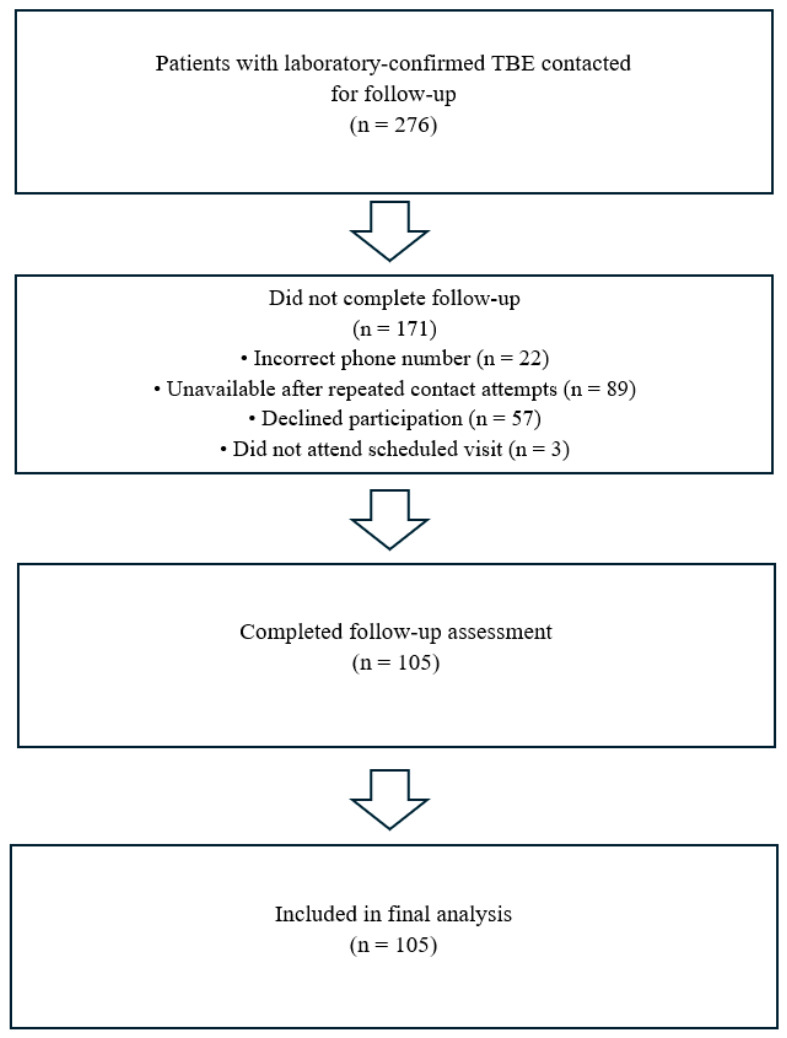
Flow diagram of patient selection and follow-up.

For the prospective follow-up phase, 276 patients with laboratory-confirmed TBE who had been hospitalized between 2018 and 2024 were contacted. Of these, 105 agreed to participate and completed the follow-up assessment. Among the remaining patients, 57 declined participation, 89 could not be reached despite repeated contact attempts, 22 had incorrect or unavailable contact information, and 3 did not attend the scheduled follow-up visit. A flow diagram summarizing participant recruitment and follow-up is provided in Figure 1.

Ethical approval was obtained from Rīga Stradiņš University Ethics Committee (NR. 2/30.11.2017 and 22-2/27/2021).

### 2.2. Data Collection

Demographic and clinical data—including age, sex, vaccination status, history of tick bite, occupation, clinical course, comorbidities, length of hospital stay, and serological findings—were obtained from medical records.

Follow-up was conducted ≥6 months after hospital discharge and involved a structured questionnaire assessing neurocognitive, subjective, and neurological sequelae, as well as a neurological examination. The actual follow-up period ranged from 6 to 57 months, with a median of 20 months and a mean of 24.7 ± 15.7 months.

### 2.3. Definitions

The diagnosis of confirmed TBE was established according to the European Centre for Disease Prevention and Control (ECDC) case definition, which requires clinical symptoms of central nervous system inflammation (such as meningitis, meningoencephalitis, or encephalomyelitis) combined with laboratory confirmation via e.g., the detection of TBE-specific IgM and IgG antibodies in serum, or TBE-specific IgM in cerebrospinal fluid.

Epidemiological exposure was defined as the patient’s self-reported risk setting or suspected activity occurring within the probable incubation period prior to symptom onset. This information was systematically extracted from acute-phase medical records and patient histories taken upon hospital admission. The exposure settings evaluated included recreational outdoor activities, forest walking, mushroom or berry picking, peridomestic exposure, and alimentary exposure (e.g., consumption of unpasteurized milk or dairy products). The exposure categories were not mutually exclusive, as patients frequently reported multiple concurrent risk activities. Patients who did not recall a specific tick bite or risk activity, or whose medical records lacked this documentation, had their exposure status classified as unknown or unspecified. These cases were retained in the overall cohort but were excluded from the denominator when calculating the specific proportions of exposure types.

Health-related quality of life and functional status were evaluated simultaneously with the patient-reported symptom questionnaires during the follow-up assessment, which occurred at least 6 months post-discharge. To assess quality of life, we utilized the EQ-5D-5L questionnaire, which evaluates five dimensions (mobility, self-care, usual activities, pain/discomfort, and anxiety/depression) on a five-level scale ranging from 1 (no problems) to 5 (extreme problems). For this study, a patient’s quality of life was defined as impaired if they reported a score of [≥2] in at least one of the five dimensions. We also recorded the score from the EQ Visual Analogue Scale (EQ VAS), which captures the patient’s self-rated overall health on a continuous scale from 0 (worst imaginable health) to 100 (best imaginable health). Concurrently, overall functional status was measured using the Karnofsky Performance Status (KPS) scale, which ranges from 100 (normal, no complaints) to 0 (dead) in decile increments. In our analysis, a KPS score of [<100] was utilized as the threshold to define the presence of functional limitations following TBE infection. For statistical evaluation, the EQ-5D-5L impairment criteria and KPS scores were treated as variables to compare the prevalence of long-term impairment across groups with different clinical severity.

Meningitis was defined as a clinical presentation consistent with viral meningitis (e.g., headache, neck stiffness, photophobia, hyperacusis, and fever) without signs of encephalitis, which was defined according to the International Encephalitis Consortium criteria [10].

Clinical form and disease severity were evaluated as separate variables, in which the former was defined according to the pattern of central nervous system involvement and categorized as meningitis or meningoencephalitis, whereas the latter referred to the intensity of acute neurological dysfunction. Acute disease severity was assessed using the *Mickienė* [11] and *Bogovič* [12] classification systems, which categorize disease as mild, moderate, or severe based on clinical manifestations and neurological involvement.

According to the *Mickienė* criteria, mild disease was defined by predominantly meningeal symptoms, including fever, headache, neck stiffness, and nausea. Moderate disease was defined by the presence of monofocal central nervous system (CNS) symptoms and/or moderate diffuse brain dysfunction. Severe disease was defined by multifocal CNS involvement and/or severe diffuse brain dysfunction [11].

According to the *Bogovič* classification, disease severity was additionally assessed using a point-based clinical scoring system that included headache, fever, vomiting, meningeal signs, tremor, paresis, urinary retention, cognitive dysfunction, disturbances of consciousness (somnolence, stupor or coma), and treatment for elevated intracranial pressure. Each symptom, or its duration when applicable, contributes to the total score. Mild disease was defined as 0–8 points, moderate disease as 9–22 points, and severe disease as >22 points [12].

The *Mickienė* and *Bogovič* classifications were used to stratify acute disease severity for analysis of post-encephalitic sequelae. Due to missing data for acute disease classification, subgroup analyses differ slightly between severity scoring systems (*Bogović n* = 52; *Mickienė n* = 51).

In this study, the “abortive” form of the disease was defined as a symptomatic TBE virus infection that remains restricted to the initial viremic (febrile) phase. Patients presenting with this form experience systemic, nonspecific symptoms—such as fever, fatigue, headache, and myalgia—but recover without developing any clinical signs or symptoms of central nervous system (CNS) involvement.

Sequelae were defined as post-encephalitic syndrome (PES) according to the Bohr criteria. PES was defined by the presence of ≥2 subjective symptoms—including headache, memory and/or concentration impairment, general fatigue, arthralgia and/or myalgia, emotional lability, sleep disturbance, or dizziness—or ≥1 objective neurological sign, including tremor, paresis, or hearing or visual impairment [13].

PES severity was classified according to the original Bohr criteria and subsequent operational definitions, as follows: mild, if two subjective symptoms fulfilling criteria for subjective symptoms were present, or moderate, if ≥3 subjective symptoms affected quality of life without requiring environmental adaptation. Severe PES was defined as the presence of ≥1 objective neurological manifestation associated with persistent post-encephalitic symptoms and clinically relevant functional impairment affecting daily activities, in accordance with the Bohr criteria [4,5].

TBE was confirmed at the reference laboratory of the Latvian Center of Infectious Diseases. Diagnosis was based on the detection of TBE virus-specific IgM and IgG antibodies in the patients’ serum, and/or TBE-specific IgM antibodies in the cerebrospinal fluid (CSF). The serological analysis was conducted using commercial enzyme-linked immunosorbent assay (ELISA) kits (EUROIMMUN Medizinische Labordiagnostika AG, Lübeck, Germany), following the manufacturer’s instructions. Results were interpreted according to the manufacturer’s cut-off values: for anti-TBEV IgM, a ratio of <0.8 was considered negative, 0.8–1.1 borderline, and ≥1.1 positive; for anti-TBEV IgG, a concentration of <16 VIEU/mL was considered negative, 16–22 VIEU/mL borderline, and >22 VIEU/mL positive. Cases with isolated borderline results were retested or excluded if confirmation could not be established.

### 2.4. Statistical Analysis

Statistical analyses were performed using IBM SPSS Statistics Version 30. Continuous variables are presented as mean ± standard deviation (SD) or median (interquartile range [IQR]), as appropriate. Categorical variables are expressed as frequencies and percentages (*n*/N, %).

Group comparisons were performed using the χ^2^ test or Fisher’s exact test for categorical variables and the independent-samples *t*-test or Mann–Whitney U test for continuous variables, as appropriate.

Associations between continuous or ordinal variables were assessed using Spearman’s rank correlation coefficient.

Binary logistic regression analysis was performed to identify factors associated with the presence of post-encephalitic syndrome (PES), in which the dependent variable was the presence or absence of PES. The results are reported as odds ratios (ORs) with 95% confidence intervals (CIs).

Ordinal logistic regression analysis was performed to evaluate predictors of sequelae severity. The dependent variable was sequelae severity, categorized as no sequelae, mild PES, and moderate PES. Independent variables considered for inclusion in the models were age, sex, comorbidity status, biphasic disease course, clinical form (meningitis versus meningoencephalitis), acute neurological manifestations, and measures of acute disease severity. Female sex, absence of comorbidities, absence of biphasic disease, and meningitis were used as reference categories for categorical variables. Results are reported as ORs, 95% CIs, and exact *p*-values.

Missing data were handled using complete-case analysis; patients with missing values for variables included in a given model were excluded from that analysis. Consequently, the sample size differed slightly between regression models.

Model fit was assessed using the likelihood ratio test, and the proportional odds assumption was evaluated using the test of parallel lines.

All statistical tests were two-tailed, and *p* < 0.05 was considered statistically significant.

## 3. Results

### 3.1. Baseline Characteristics

A total of 105 patients were included, 58 (55.2%) of which were female. The mean age was 51.4 ± 13.6 years (range 20–85 years), with the largest age group being 41–50 years (32/105 [30.5%]). The baseline demographic and clinical characteristics were comparable between the male and female patients, including age (*p* = 0.434), comorbidity status (*p* = 0.478), clinical form of disease (*p* = 0.529), biphasic course (*p* = 0.835), and vaccination status (*p* = 0.916) (Supplementary Table S1).

Most patients were from the Vidzeme (62/105 (59.0%)), Kurzeme (35/105 (33.3%)), and Zemgale regions (5/105 (4.8%)). A history of tick bites was not reported by 39 (37.1%) of the patients, and concomitant Lyme disease was present in 9/105 (8.5%).

Exposure most frequently occurred during recreational outdoor activities, particularly forest walking (37/103, 35.9%) and mushroom or berry picking (19/103, 18.4%). Peridomestic exposure accounted for 12/103 cases (11.7%). Two patients had missing exposure data.

Most patients were unvaccinated (98/105, 93.3%), and 57/105 (54.3%) had at least one comorbidity.

Detailed characteristics of the patients are presented in Table 1.

### 3.2. Clinical Course

Disease onset occurred predominantly during the high-tick-activity season (May–October). Overall, the highest numbers of cases were recorded in July (36/105, 34.3%) and August (24/105, 22.9%). The analysis by calendar year demonstrated a broadly consistent seasonal pattern throughout 2018–2024, with most cases occurring between June and September (Supplementary Table S2). The annual peak month varied between years (June–September), but July accounted for the highest number of cases in several study years (2020, 2021, and 2023). A biphasic course was observed in 88/105 (83.8%) patients.

Fever (100/105, 95.2%) and headache (92/105, 87.6%) were the most common symptoms, with headache being more frequent among females than males (55/58 [94.8%] vs. 37/47 [78.7%], *p* = 0.013). Other common symptoms included fatigue (90/105, 85.7%) and arthralgia/myalgia (57/105, 54.3%).

According to the *Mickienė* criteria, among 101 patients with available data, 71/101 (70.3%) had a mild acute disease course, 23/101 (22.8%) had a moderate course, and 7/101 (6.9%) had a severe course. Disease severity was unknown for four patients.

Using the *Bogovič* classification, 71/105 (67.6%), 26/105 (24.8%), and 5/105 (4.8%) patients were classified as having mild, moderate, and severe acute diseases, respectively.

Meningeal signs were present in 66 (62.9%) of the patients and were more frequent in females (*p* < 0.001).

Neurological manifestations were mainly observed in the patients with meningoencephalitis, with tremor being the most frequent finding (25/105, 23.8%), followed by ataxia, impaired consciousness, and paresis.

The median hospital stay was 10 days (IQR 8–12), with no significant differences between clinical forms.

Lumbar puncture was performed in 98/105 (94.1%) patients, showing that pleocytosis was present in all but one case. Cerebrospinal fluid (CSF) leukocyte counts tended to be higher in meningoencephalitis than in meningitis cases (median 102 vs. 43 cells/µL; *p* = 0.053). However, no association was observed between the CSF leukocyte count and sequelae severity.

The subjective, neurological, and other symptoms recorded in the acute phase of the disease are presented in Table 2, Table 3 and Table 4.

### 3.3. Sequelae

Sequelae were observed in 52/105 patients (49.5%); however, subgroup analyses according to the *Mickienė* classification included 51 patients due to missing data. Sequelae were more frequent in patients with meningoencephalitis than in those with meningitis (18/25 [72.0%] vs. 33/77 [42.9%]).

According to the Bohr criteria for the severity of post-encephalitic syndrome (PES), most patients with PES had mild sequelae (42/52, 80.8%), and 10/52 (19.2%) had moderate sequelae, with no cases of severe sequelae observed.

The most common sequelae were difficulty concentrating (33/52 [63.5%]), fatigue (29/52 [55.8%]), and sleep disturbances (21/52 [40.4%]). Emotional lability was associated with more severe acute disease (*p* = 0.027).

Neurological sequelae included tremor (23/52 [44.2%]), vertigo (11/52 [21.2%]), and hearing impairment (5/52 [9.8%]). Paresis occurred only in patients with moderate or severe acute disease (*p* = 0.004). Although objective neurological signs were present in some patients during follow-up, none fulfilled the full Bohr criteria for severe post-encephalitic syndrome, which requires persistent symptoms associated with clinically relevant functional impairment. Therefore, no cases of severe PES were identified in the cohort.

Most patients achieved favorable functional outcomes according to the Karnofsky Performance Status Scale. According to the EQ-5D-5L version 3 (V3) scale, quality of life was affected in 17/52 patients (32.7%) with post-encephalitic syndrome.

The post-encephalitic sequelae recorded, according to acute disease severity as defined by the *Bogović* or *Mickienė* classification, are presented in Table 5 and Table 6. The neurocognitive, subjective, and neurological sequelae stratified by age and sex are presented in Table 7.

### 3.4. Factors Associated with Sequelae

Older age was associated with the presence and severity of sequelae. The patients with sequelae were older (*p* = 0.043), and age correlated with disability at discharge. In the multivariable analysis, age remained independently associated with greater sequelae severity (OR = 1.045 per year, *p* = 0.003), whereas sex, hypertension, and other comorbidities were not significant predictors.

Biphasic disease was not significantly associated with sequelae severity, although a non-significant trend toward lower severity was observed (OR = 0.45, 95% CI 0.17–1.23; *p* = 0.120).

The clinical form was associated with the severity of the sequelae in the overall ordinal regression model (*p* = 0.001), but the adjusted pairwise comparisons did not reveal significant differences; a trend towards greater severity was observed in patients with meningoencephalitis.

The severity of the disease, assessed using the *Mickienė* and *Bogovič* classifications, was significantly associated with the development of PES. Moderate and severe cases showed a higher frequency of PES compared with mild cases under both classifications (*Mickienė*: 21/30 (70.0%) and 11/16 (68.8%) vs. 14/34 (41.2%), χ^2^(2) = 6.81, *p* = 0.033; *Bogovič*: 20/29 (69.0%) and 4/5 (80.0%) vs. 29/71 (40.8%), χ^2^(2) = 8.03, *p* = 0.018). However, neither classification independently predicted sequelae severity after adjustment, although a non-significant trend was observed for the *Bogović* score (*p* = 0.100).

The length of hospital stay was not associated with sequelae severity (OR ≈ 1.06 per day; *p* = 0.199).

Acute-phase neurological manifestations were associated with outcomes. Specifically, the presence of objective neurological signs was associated with a higher frequency of PES (38/52 (73.1%) vs. 24/53 (45.3%); χ^2^(1) = 3.97, *p* = 0.046). Paresis was strongly associated with greater sequelae severity (OR ≈ 21.7; *p* = 0.002), whereas meningeal syndrome was not (*p* = 0.941; overall model *p* = 0.004). Tremor was also associated with increased severity (OR ≈ 3.5; *p* = 0.019), while ataxia and somnolence were not significant predictors (overall model *p* < 0.001).

Among neuropsychiatric symptoms, emotional lability was associated with a higher frequency of PES (43/52 (82.7%) vs. 23/53 (43.4%); χ^2^(1) = 8.75, *p* = 0.003; OR = 6.14), while sleep disturbances showed a similar association (37/52 (71.2%) vs. 23/53 (43.4%); χ^2^(1) = 6.63, *p* = 0.010).

## 4. Discussion

This study demonstrates that nearly half of the adult patients (52/105 [49.5%]) hospitalized with tick-borne encephalitis developed long-term sequelae, most commonly mild neurocognitive impairment. The prevalence of post-encephalitic syndrome in this study lies at the upper range of previously reported estimates, likely reflecting systematic follow-up and the inclusion of subjective and neurological outcomes.

A key finding is that age was the only independent predictor of sequelae severity, consistent with previous studies reporting poorer neurological recovery in older patients.

In our multivariate analysis, older age was identified as the only independent predictor of long-term sequelae severity. However, this finding must be interpreted with caution. Given the relatively small sample size and the missing data for certain acute-phase severity classifications, our regression models had limited statistical power and may be unstable. Consequently, the lack of statistical significance for other clinical and demographic variables in our cohort does not definitively rule out their prognostic relevance. It is highly plausible that factors such as the initial clinical form, acute disease severity, or the presence of specific comorbidities also influence the long-term outcomes of TBE, even though our study lacked the power to detect these independent associations. Therefore, while advancing age clearly contributes to the risk of severe sequelae, our results should not be interpreted as evidence that other variables are non-predictive. Larger, prospective studies are required to build more comprehensive and stable prognostic models.

In contrast, sex, comorbidities, biphasic disease, and length of hospital stay were not associated with long-term outcomes, suggesting the limited prognostic value of these variables when considered in isolation. Similarly, cerebrospinal fluid parameters did not show predictive value, supporting previous observations that routine inflammatory markers during the acute phase do not reliably reflect long-term neurological recovery.

Although meningoencephalitis was associated with higher crude sequelae rates, this association did not persist after adjustment. This finding suggests that the clinical phenotype alone may be insufficient to predict the long-term outcome when controlling for age and acute neurological involvement.

Importantly, acute neurological manifestations—particularly paresis and tremor—were strongly associated with worse long-term outcomes, indicating that focal neurological deficits during the acute phase may better capture the risk of persistent dysfunction than global severity classifications. Although the *Mickienė* and *Bogovič* scoring systems correlated with sequelae in univariate analyses, neither independently predicted outcomes after adjustment, highlighting limitations of current severity grading systems for prognostic stratification.

Despite a high prevalence of subjective symptoms, most patients maintained good functional status, underscoring a discrepancy between patient-reported outcomes and functional scales. This supports previous findings that post-encephalitic syndrome carries with it a substantial burden of “invisible disability,” particularly cognitive impairment and fatigue.

## 5. Limitations

Our study has several notable limitations that must be considered when interpreting the findings. First, the study used a hybrid design that involved the retrospective extraction of acute-phase clinical data, which inherently introduces issues with missing or incomplete medical documentation. Furthermore, our cohort was restricted to patients who required hospitalization for TBE, thereby excluding milder, non-hospitalized cases. This restriction, combined with the absence of a non-TBE control group, limits the generalizability of our findings and may lead to an overestimation of the overall frequency of severe long-term sequelae in the broader TBE-infected population. There is also potential for selection bias during the prospective phase, as patients experiencing persistent, bothersome symptoms may have been more motivated to participate in the follow-up than those who had fully recovered.

Second, the relatively small total sample size (*n* = 105) restricted our analytical power. This was compounded by missing clinical data, which significantly reduced the number of patients who could be accurately categorized using the *Bogovič* and *Mickienė* acute disease severity classifications. Consequently, there were very few patients in the moderate and severe acute subcategories. As a result of these small numbers, our multivariate regression models may be unstable; therefore, our predictive estimates—such as the independent prognostic value of age—should be interpreted with caution and validated in larger, multicenter cohorts.

Third, our assessment of long-term post-encephalitic syndrome (PES) was primarily based on patient-reported outcome measures (PROMs). While highly relevant for capturing the patient-perceived burden of disease, relying on subjective symptoms introduces recall and perception biases, particularly in the absence of standardized, objective neuropsychological and neurological testing to clinically confirm these deficits. Moreover, the timing of the follow-up assessments (defined broadly as ≥6 months post-discharge) was somewhat heterogeneous, which complicates the strict temporal standardization of the long-term outcomes.

Finally, a subset of our cohort presented with concomitant Lyme borreliosis. Although these cases were documented, tick-borne coinfections are a potential confounding factor, as the long-term subjective and neurological symptoms of Lyme disease—particularly Lyme neuroborreliosis—can clinically overlap with TBE sequelae.

## 6. Conclusions

Tick-borne encephalitis is associated with a substantial burden of long-term sequelae, particularly neurocognitive symptoms, with increasing age being the main determinant of worse outcomes. Further large-scale longitudinal studies are needed to improve prognostic assessment and guide clinical management strategies.

## Figures and Tables

**Table 1:** Baseline characteristics of patients with tick-borne encephalitis (*n* = 105).

Characteristic	*n*	%
Sex		
Female	58	55.2
Male	47	44.8
Age		
Mean ± SD (years)	51.37 ± 13.58	-
Range (years)	20–85	-
Age groups		
20–30	3	2.9
31–40	20	19.0
41–50	32	30.5
51–60	22	21.0
61–70	19	18.1
71–80	7	6.7
81–90	2	1.9
Tick bite noticed		
Yes	66	62.9
No	39	37.1
Vaccination status		
Unvaccinated	98	93.3
Incomplete vaccination	7	6.7
Comorbidities		
Present	57	54.3
Absent	48	45.7
Concomitant Lyme disease	9	8.5
Cardiovascular diseases	25	23.8
Endocrine/metabolic diseases	13	12.4
Headache disorders	4	3.8
Hepatic diseases	3	2.9
Malignancies	4	3.8
Psychiatric disorders	2	1.9

**Table 2:** Subjective symptoms recorded in the acute phase of the disease. Data are presented as *n* (%).

Variable	Total (*n* = 105)	Male (*n* = 47)	Female (*n* = 58)	*p*-Value *	Meningitis (*n* = 77)	Meningoencephalitis (*n* = 25)	*p*-Value **
Headache	92 (87.6%)	37 (78.7%)	55 (94.8%)	0.013	67 (87.0%)	22 (88.0%)	1.000
General fatigue	90 (85.7%)	41 (87.2%)	49 (85.5%)	0.689	67 (87.0%)	21 (84.0%)	0.742
Arthralgia/ myalgia	57 (54.3%)	26 (55.3%)	31 (53.4%)	0.848	45 (58.4%)	11 (44.0%)	0.251
Memory impairment	14 (13.3%)	5 (10.6%)	9 (15.5%)	0.465	7 (9.1%)	6 (24.0%)	0.080
Difficulty concentrating	39 (37.1%)	15 (31.9%)	24 (41.4%)	0.318	28 (36.4%)	10 (40.0%)	0.813
Emotional lability	17 (16.2%)	7 (14.9%)	10 (17.2%)	0.745	13 (16.9%)	4 (16.0%)	1.000
Insomnia	25 (23.8%)	10 (21.3%)	15 (25.9%)	0.583	19 (24.7%)	6 (24.0%)	1.000
Somnolence	34 (32.4%)	10 (21.3%)	24 (41.4%)	0.036	21 (27.3%)	13 (52.0%)	0.029

*p*-value *—comparison between males and females (χ^2^ test or Fisher’s exact test). *p*-value **—comparison between meningitis and meningoencephalitis. Subgroup analyses exclude three patients with abortive disease form.

**Table 3:** Neurological signs and symptoms recorded in the acute phase of the disease, stratified by sex and clinical form.

Variable	Total (*n* = 105)	Male (*n* = 47)	Female (*n* = 58)	*p*-Value *	Meningitis (*n* = 77)	Meningoencephalitis (*n* = 25)	*p*-Value **
Visual impairment	9 (8.7%)	2 (4.3%)	7 (12.3%)	0.179	6 (7.9%)	3 (12.0%)	0.686
Impaired consciousness	6 (5.7%)	3 (6.4%)	3 (5.2%)	1.000	1 (1.3%)	5 (20.0%)	0.003
Ataxia	6 (5.7%)	1 (2.1%)	5 (8.6%)	0.221	0	6 (24.0%)	<0.001
Paresis	5 (4.9%)	1 (2.1%)	4 (7.1%)	0.372	0	5 (20.0%)	<0.001
Cranial neuropathies	2 (1.9%)	1 (2.1%)	1 (1.7%)	1.000	1 (1.3%)	1 (4.0%)	0.432
Tremor	25 (23.8%)	9 (19.1%)	16 (27.6%)	0.313	1 (1.3%)	24 (96.0%)	<0.001
Dysphagia	1 (1.0%)	1 (2.1%)	0	0.448	0	1 (4.0%)	0.245
Dysphonia/dysarthria	2 (1.9%)	2 (4.3%)	0	0.198	0	2 (8.0%)	0.058
Dysphasia	2 (1.9%)	2 (4.3%)	0	0.198	0	2 (8.0%)	0.058
Meningeal signs	66 (62.9%)	20 (42.6%)	46 (79.3%)	<0.001	47 (61.0%)	19 (76.0%)	0.230
Sensory disturbance	3 (2.9%)	0	3 (5.2%)	0.251	0	3 (12.0%)	0.013

Data are presented as *n* (%). *p*-value *—comparison between males and females (χ^2^ test or Fisher’s exact test). *p*-value **—comparison between meningitis and meningoencephalitis. Subgroup analyses exclude three patients with abortive disease form.

**Table 4:** Other clinical symptoms recorded in the acute phase of the disease, stratified by sex and clinical form.

Variable	Total (*n* = 105)	Male (*n* = 47)	Female (*n* = 58)	*p*-Value *	Meningitis (*n* = 77)	Meningoencephalitis (*n* = 25)	*p*-Value **
Fever	100 (95.2%)	46 (97.9%)	54 (93.1%)	0.377	75 (97.4%)	22 (88.0%)	0.093
Biphasic	88 (83.8%)	39 (83.0%)	49 (84.5%)	1.000	66 (85.7%)	21 (84.0%)	1.000
disease							
Psychotic	4 (3.8%)	1 (2.1%)	3 (5.2%)	0.626	2 (2.6%)	2 (8.0%)	0.251
symptoms							
Respiratory problems	2 (1.9%)	1 (2.1%)	1 (1.7%)	1.000	1 (1.3%)	1 (4.0%)	0.432
Defecation problems	1 (1.0%)	0	1 (1.7%)	1.000	0	1 (4.0%)	0.245
Nausea	51 (48.6%)	13 (27.7%)	38 (65.5%)	<0.001	37 (48.1%)	14 (56.0%)	0.646
Diarrhea/abdominal pain	16 (15.2%)	4 (8.5%)	12 (20.7%)	0.084	10 (13.0%)	5 (20.0%)	0.515
Vomiting	29 (27.6%)	7 (14.9%)	22 (37.9%)	0.015	23 (29.9%)	6 (24.0%)	0.621

Data are presented as *n* (%). *p*-value *—comparison between males and females (χ^2^ test or Fisher’s exact test). *p*-value **—comparison between meningitis and meningoencephalitis. Subgroup analyses exclude three patients with abortive disease form.

**Table 5:** Post-encephalitic sequelae recorded according to acute disease severity as defined by *Bogović* classification.

Outcome	Total (*n* = 52)	Mild (*n* = 29)	Moderate (*n* = 18)	Severe (*n* = 4)	*p*-Value
Neurocognitive sequelae					
Difficulty concentrating	32 (62.7%)	19 (65.5%)	11 (61.1%)	2 (50.0%)	0.750
Emotional lability	22 (43.1%)	8 (27.6%)	11 (61.1%)	3 (75.0%)	0.027
Sleep disturbance	21 (41.2%)	13 (44.8%)	6 (33.3%)	2 (50.0%)	0.679
Subjective symptoms					
General fatigue	29 (56.9%)	13 (44.8%)	13 (72.2%)	3 (75.0%)	0.140
Headache	16 (31.4%)	9 (31.0%)	6 (33.3%)	1 (25.0%)	1.000
Arthralgia/myalgia	11 (21.6%)	6 (20.7%)	5 (27.8%)	0	0.671
Neurological sequelae					
Vertigo	11 (21.6%)	6 (20.7%)	5 (27.8%)	0	0.671
Visual impairment	2 (3.9%)	1 (3.4%)	1 (5.6%)	0	1.000
Hearing impairment	5 (9.8%)	2 (6.9%)	2 (11.1%)	1 (25.0%)	0.327
Paresis	4 (7.8%)	0 (0.0%)	2 (11.1%)	2 (50.0%)	0.004
Tremor	23 (45.1%)	11 (37.9%)	11 (61.1%)	1 (25.0%)	0.256

*p*-values were calculated using Pearson’s Chi-square or Fisher’s exact test when expected counts were <5. Mild—patients with mild acute disease; moderate—patients with moderate acute disease; severe—patients with severe acute disease.

**Table 6:** Post-encephalitic sequelae recorded according to acute disease severity as defined by Mickiene classification.

Outcome	Total (*n* = 51)	Mild (*n* = 30)	Moderate (*n* = 16)	Severe (*n* = 5)	*p*-Value
Neurocognitive sequelae					
Difficulty concentrating	30 (58.8%)	20 (66.7%)	9 (56.3%)	3 (60.0%)	0.760
Emotional lability	22 (43.1%)	9 (30.0%)	9 (56.3%)	4 (80.0%)	0.057
Sleep disturbance	21 (41.2%)	14 (46.7%)	5 (31.3%)	2 (40.0%)	0.648
Subjective symptoms					
General fatigue	29 (56.9%)	14 (46.7%)	11 (68.8%)	4 (80.0%)	0.254
Headache	16 (31.4%)	9 (30.0%)	6 (37.5%)	1 (20.0%)	0.821
Arthralgia/myalgia	11 (21.6%)	6 (20.0%)	5 (31.3%)	0	0.411
Neurological sequelae					
Vertigo	11 (21.6%)	7 (23.3%)	3 (18.8%)	1 (20.0%)	1.000
Visual impairment	2 (3.9%)	1 (3.3%)	1 (6.3%)	0	1.000
Hearing impairment	5 (9.8%)	2 (6.7%)	2 (12.5%)	1 (20.0%)	0.468
Paresis	4 (7.8%)	0	2 (12.5%)	2 (40.0%)	0.007
Tremor	23 (45.1%)	11 (36.7%)	10 (62.5%)	2 (40.0%)	0.233

Disease severity was not known for one patient.

**Table 7:** Neurocognitive, subjective, and neurological sequelae stratified by age and sex among patients with post-encephalitic sequelae (*n* = 52).

Outcome	Total (*n* = 52)	Age 20–40 (*n* = 9)	Age 41–60 (*n* = 27)	Age ≥ 61 (*n* = 16)	*p*-Value *	Male (*n* = 19)	Female (*n* = 33)	*p*-Value **
Neurocognitive sequelae								
Difficulty concentrating	33 (63.5%)	8 (88.9%)	14 (51.9%)	11 (68.8%)	0.113	10 (52.6%)	23 (69.7%)	0.246
Emotional lability	22 (42.3%)	3 (33.3%)	11 (40.7%)	8 (50.0%)	0.804	6 (31.6%)	16 (48.5%)	0.261
Sleep disturbance	21 (40.4%)	4 (44.4%)	9 (33.3%)	8 (50.0%)	0.600	5 (26.3%)	16 (48.5%)	0.149
Subjective symptoms								
General fatigue	29 (55.8%)	5 (55.6%)	17 (63.0%)	7 (43.8%)	0.488	12 (63.2%)	17 (51.5%)	0.563
Headache	17 (32.7%)	3 (33.3%)	8 (29.6%)	6 (37.5%)	0.922	7 (36.8%)	10 (30.3%)	0.761
Arthralgia/myalgia	11 (21.2%)	0	6 (22.2%)	5 (31.3%)	0.274	4 (21.1%)	7 (21.2%)	1.000
Neurological sequelae								
Vertigo	11 (21.2%)	1 (11.1%)	4 (14.8%)	6 (37.5%)	0.232	4 (21.1%)	7 (21.2%)	1.000
Visual impairment	3 (5.8%)	1 (11.1%)	1 (3.7%)	1 (6.3%)	0.746	1 (5.3%)	2 (6.1%)	1.000
Hearing impairment	5 (9.6%)	0	2 (7.4%)	3 (18.8%)	0.371	3 (15.8%)	2 (6.1%)	1.000
Paresis	4 (7.7%)	1 (11.1%)	2 (7.4%)	1 (6.3%)	1.000	0	4 (12.1%)	0.284
Tremor	23 (44.2%)	2 (22.2%)	12 (44.4%)	9 (56.3%)	0.294	10 (52.6%)	13 (39.4%)	0.397

*p*-value *—comparison between age groups (χ^2^ test or Fisher’s exact test). *p*-value **—comparison between males and females. Analyses include only patients who developed post-encephalitic sequelae (*n* = 52).

## Data Availability

The data presented in this study are available on request from the corresponding author. The data are not publicly available due to [reason-legal restrictions].

## Supplementary Materials

The following supporting information can be downloaded at: https://www.mdpi.com/article/10.3390/pathogens15070672/s1, Table S1: Baseline demographic and clinical characteristics stratified by sex (*n* = 105); Table S2: Monthly distribution of tick-borne encephalitis cases by year of disease onset among study participants, Latvia, 2018–2024 (*n* = 105).
