# Supplementary material for: Long-Term Outcomes in Adult Patients with Tick-Borne Encephalitis in Latvia
Source: Pathogens. 2026 Jun 25;15(7):672. doi: 10.3390/pathogens15070672 (PMC13414771; doi:10.3390/pathogens15070672)
Supplement: Supplementary file 1 [file pathogens-15-00672-s001.zip › pathogens-4326855-supplementary.pdf]

Supplementary Table S1. Baseline demographic and clinical characteristics stratified by sex (n = 105).

| Characteristic | Male (n = 47) | Female (n = 58) | p-value |
|---|---|---|---|
| **Age, years, mean ± SD** | 50.2 ± 13.0 | 52.3 ± 14.1 | 0.434 |
| **Comorbidities, n (%)** | 27 (58.7%) | 30 (51.7%) | 0.478 |
| **Vaccination status, n (%)** | 3 (6.4%) | 4 (6.9%) | 0.916 |
| **Clinical form, n (%)** | | | 0.529 |
| **Meningitis** | 37 (78.7%) | 40 (69.0%) | |
| **Meningoencephalitis** | 9 (19.1%) | 16 (27.6%) | |
| **Abortive form** | 1 (2.1%) | 2 (3.4%) | |
| **Biphasic disease, n (%)** | 39 (83.0%) | 49 (84.5%) | 0.835 |

Supplementary Table S2. Monthly distribution of tick-borne encephalitis cases by year of disease onset among study participants, Latvia, 2018–2024 (n = 105).

| Year | Mar | Apr | May | Jun | Jul | Aug | Sep | Oct | Total |
|---|---|---|---|---|---|---|---|---|---|
| **2018** | 0 | 0 | 1 | 2 | 2 | 1 | 3 | 1 | 10 |
| **2019** | 0 | 0 | 2 | 0 | 5 | 6 | 2 | 0 | 15 |
| **2020** | 2 | 0 | 3 | 4 | 9 | 3 | 1 | 1 | 23 |
| **2021** | 0 | 0 | 0 | 1 | 3 | 1 | 0 | 0 | 5 |
| **2022** | 0 | 1 | 1 | 7 | 4 | 4 | 4 | 2 | 23 |
| **2023** | 1 | 0 | 2 | 4 | 12 | 8 | 0 | 0 | 27 |
| **2024** | 0 | 0 | 0 | 0 | 1 | 1 | 0 | 0 | 2 |
| **Total** | **3** | **1** | **9** | **18** | **36** | **24** | **10** | **4** | **105** |

Data are presented as mean ± standard deviation or number (percentage). p-values were calculated using independent-samples t-test for continuous variables and Chi-square or Fisher’s exact test for categorical variables, as appropriate.
